# Genetic susceptibility and neuroprotective gene therapy of normal tension glaucoma: a review

**DOI:** 10.3389/fmed.2026.1950490

**Published:** 2026-09-28

**Authors:** Xiaoyu Liu, Dadong Jia, Hao Hu, Liang Liang

**Affiliations:** Department of Ophthalmology, The Second People’s Hospital Affiliated to China Three Gorges University, Yichang Second People’s Hospital, Yichang, China

**Keywords:** adeno-associated virus, autophagy, circadian rhythm, gene therapy, mitochondrial dysfunction, normal-tension glaucoma, optic nerve protection, vascular dysregulation

## Abstract

Normal-tension glaucoma (NTG) is a distinct form of glaucomatous optic neuropathy characterized by progressive retinal ganglion cell (RGC) degeneration and visual field loss despite intraocular pressure (IOP) remaining within the statistically normal range. Although IOP reduction remains the only established disease-modifying strategy, continued disease progression despite adequate IOP control highlights the importance of IOP-independent mechanisms. Increasing evidence implicates vascular dysregulation, mitochondrial dysfunction, oxidative stress, impaired autophagy, neuroinflammation, axonal transport abnormalities, and circadian dysregulation in increased RGC vulnerability. Genetic studies have identified *OPTN* and *TBK1* as the most strongly supported NTG-associated susceptibility genes, whereas many other candidate genes, including *MYOC*, *SARM1*, *DLK*, *BDNF*, *NOS3*, *KLF2* and *PTGDS*, are shared with other glaucoma subtypes or remain supported primarily by experimental or mechanistic evidence. These interconnected processes converge on mitochondrial impairment, axonal degeneration, and RGC death, providing potential therapeutic targets beyond conventional IOP lowering. This review integrates the vascular, mitochondrial, and neuronal mechanisms underlying NTG, distinguishes NTG-associated molecular factors from pathways shared across glaucoma subtypes, and summarizes emerging gene- and nucleic-acid-based therapeutic strategies. By linking evidence-based mechanisms with emerging molecular technologies, this review provides a framework for mechanism-based and precision-oriented therapies that may complement established IOP-lowering treatment for NTG.

## 1 Introduction

Normal-tension glaucoma (NTG) represents a distinct phenotype within the primary open-angle glaucoma (POAG) spectrum, characterized by progressive glaucomatous optic nerve damage, retinal nerve fiber layer thinning, and retinal ganglion cell (RGC) loss despite IOP remaining within the statistically normal range (1, 2). Although IOP reduction remains the cornerstone of NTG management, including topical medications, selective laser trabeculoplasty, and surgical interventions, disease progression may occur despite substantial IOP lowering (3–6). Moreover, a clinically normal IOP does not necessarily reflect cumulative 24-h IOP exposure, as transient IOP fluctuations, nocturnal changes, systemic blood-pressure variation, and impaired vascular autoregulation may collectively affect the mechanical and metabolic environment of the optic nerve head (2, 7).

These observations indicate that IOP alone cannot fully account for NTG pathogenesis and progression. Increasing genetic and molecular evidence suggests that NTG arises from the interaction between genetic susceptibility and systemic or environmental stressors, converging on interconnected processes including vascular dysregulation, mitochondrial dysfunction, oxidative stress, impaired autophagy and protein/organelle homeostasis, neuroinflammation, axonal degeneration, and circadian dysregulation (7–10).

Accordingly, this review focuses on the major non-IOP-dependent mechanisms and genetic determinants that may underlie the heightened vulnerability of retinal RGCs in NTG. We further evaluate emerging approaches informed by these mechanisms, including non-IOP-dependent neuroprotection, molecular phenotyping, and genotype-guided therapy, and discuss their potential integration into a precision medicine framework for NTG.

## 2 Genetic susceptibility and pathogenic molecular pathways underlying NTG

### 2.1 Vascular dysregulation and ONH impaired impaired perfusion

Vascular dysregulation and impaired optic nerve head (ONH) perfusion are important contributors to NTG pathogenesis. Given the high metabolic demands of RGCs and their axons, impaired vascular autoregulation may increase ONH susceptibility to fluctuations in systemic blood pressure and IOP, consistent with clinical evidence of reduced ONH blood flow and impaired autoregulatory responses in NTG (9).

At the molecular level, vascular homeostasis depends on the balance between vasodilatory NO/NOS3 signaling and vasoconstrictive ET-1/EDNRA signaling. Alterations in NOS3 may impair endothelial-dependent vasodilation, whereas *EDNRA* polymorphisms have been associated with NTG susceptibility and may promote ONH hypoperfusion, particularly during nocturnal blood-pressure dipping, thereby inducing metabolic and oxidative stress in RGCs (11–14). *MYOC*, *LTBP2*, and *LOXL1* primarily regulate ECM organization, tissue biomechanics, and IOP homeostasis; their potential effects on vascular compliance and adaptation to hemodynamic stress may modify vascular and tissue resilience in NTG. However, direct evidence supporting an IOP-independent role for these genes in NTG remains limited, and they should therefore be considered potential modifiers rather than established NTG-specific determinants (14–16).

Beyond canonical vasoactive pathways, genes involved in endothelial mechanosensing and extracellular matrix (ECM) homeostasis may further influence vascular susceptibility in NTG. *KLF2* is a flow-responsive transcription factor induced by endothelial shear stress and is a central regulator of vascular homeostasis. *KLF2* promotes an anti-inflammatory, antioxidative, and vasoprotective endothelial phenotype and contributes to the maintenance of endothelial responsiveness to changes in blood flow. Impaired *KLF2*-dependent shear-stress signaling could therefore weaken vascular adaptation to fluctuations in perfusion pressure, potentially increasing ONH susceptibility to hypoperfusion and ischemic stress. Because this mechanism directly links mechanical forces to endothelial function, *KLF2* represents a particularly relevant candidate for the IOP-independent vascular component of NTG (15).

### 2.2 Mitochondrial dysfunction and oxidative stress

Mitochondrial dysfunction and oxidative stress are important contributors to RGC vulnerability in NTG. Given the high energetic demands of RGCs and their axons, disturbances in mitochondrial biogenesis, mitophagy, antioxidant defense, mitochondrial dynamics, and oxidative phosphorylation may compromise mitochondrial homeostasis and increase susceptibility to degeneration, particularly under impaired ONH perfusion (17).

Mitochondrial biogenesis is coordinated by the PGC-1*α* (PPARGC1A)–NRF1/NRF2–TFAM axis, which regulates mitochondrial renewal, mtDNA maintenance, and respiratory capacity (18–20)), whereas PINK1–PRKN-mediated mitophagy removes damaged mitochondria and preserves mitochondrial quality (7, 21, 22). In parallel, NRF2 (NFE2L2)-dependent antioxidant signaling maintains redox homeostasis through cytoprotective pathways including HO-1, NQO1, glutathione metabolism, and superoxide dismutases; its impairment may promote ROS accumulation and oxidative damage to proteins, lipids, and mtDNA (23). Mitochondrial fusion and fission, mediated by *MFN1*, *MFN2*, *OPA1*, and *DRP1* (*DNM1L*), further regulate mitochondrial distribution and function, with their disruption potentially impairing axonal energy supply and RGC integrity (24, 25).

Overall, mitochondrial dysfunction in NTG reflects disruption of an interconnected homeostatic network rather than a defect in a single gene. However, evidence for most of these pathways derives from experimental glaucoma or broader neurodegenerative models, and direct NTG-specific genetic evidence remains limited. They should therefore be considered potential modifiers of RGC vulnerability, whose interaction with impaired ONH perfusion may contribute to IOP-independent neurodegeneration in NTG (2, 26).

### 2.3 Autophagy, neuroinflammation, and axonal transport dysfunction

Emerging evidence suggests that impaired autophagy, mitophagy, neuroinflammation, and axonal transport form an interconnected network contributing to RGC degeneration in NTG. Genetic alterations in *OPTN* and *TBK1* may disrupt autophagic flux and selective mitochondrial clearance. In particular, the NTG-associated *OPTN E50 K* variant has been linked to impaired autophagy–lysosomal degradation, whereas *TBK1* alterations may compromise *OPTN*-dependent autophagy and mitophagy (27, 28). Defective mitochondrial clearance can promote ROS and DAMP accumulation, thereby activating microglia and astrocytes through TLR4/MyD88/NF-*κ*B and NLRP3 inflammasome signaling and establishing a feed-forward cycle of mitochondrial stress and neuroinflammation (7, 21, 29, 30). These processes may further interact with axonal transport and cytoskeletal dysfunction, which are particularly detrimental to RGCs because of their long axons. Glaucoma-associated genes, including *OPTN*, *KIF5A*, and *SRBD1*, may contribute to intracellular trafficking and axonal integrity, while impaired microtubule-dependent transport can disrupt mitochondrial distribution and retrograde neurotrophic signaling. Thus, defects in autophagy, neuroinflammatory regulation, and axonal transport may mutually reinforce one another, ultimately promoting axonal degeneration and RGC loss despite normal-range IOP (27, 28, 31, 32).

Collectively, these findings support a convergent autophagy–neuroinflammation–axonal transport axis in NTG, in which genetic susceptibility and chronic cellular stress interact to compromise mitochondrial quality control, innate immune homeostasis, and axonal integrity. Dysfunction at one level may propagate to the others, ultimately promoting axonal degeneration and RGC loss despite IOP remaining within the statistically normal range. This interconnected network may therefore represent an important molecular interface linking genetic susceptibility to progressive, IOP-independent neurodegeneration in NTG (28, 33, 34).

### 2.4 Circadian dysregulation and RGC vulnerability

Recent transcriptomic analyses of human trabecular meshwork (TM) tissues have identified multiple circadian rhythm-related differentially expressed genes, with PTGDS emerging as a potential hub associated with immune-cell infiltration and TGF-*β*2-related fibrotic remodeling. Circadian disruption, potentially involving altered melatonin signaling, may promote PTGDS-associated inflammatory responses and tissue remodeling, thereby contributing to impaired aqueous humor outflow (35). Beyond its potential role in IOP regulation, circadian dysregulation may interact with mitochondrial quality control, autophagy, oxidative stress, and neuroinflammation, while these processes may further impair endothelial NO signaling and vascular autoregulation. Consequently, circadian dysfunction may exacerbate ONH hypoperfusion and increase RGC susceptibility, potentially linking vascular dysfunction, mitochondrial stress, and neuroinflammation in NTG (35, 36).

At the cellular level, intrinsically photosensitive retinal ganglion cells (ipRGCs) may provide an additional link between glaucomatous neurodegeneration and circadian dysregulation. Through melanopsin (OPN4)-mediated non-image-forming photoreception, ipRGCs transmit photic signals to the suprachiasmatic nucleus (SCN) via the retinohypothalamic tract, contributing to circadian photoentrainment and the regulation of sleep and melatonin secretion. Structural or functional impairment of ipRGCs may therefore reduce photic input to the SCN and promote circadian desynchronization (37, 38). Importantly, functional impairment should be distinguished from complete cell loss, as altered melanopsin signaling, dendritic architecture, intrinsic photosensitivity, or retinal input may compromise ipRGC function before substantial neuronal degeneration. Experimental and clinical studies in glaucoma further support alterations in ipRGC-related responses and SCN connectivity, suggesting that progressive RGC/ipRGC dysfunction may precede structural degeneration and impair circadian entrainment (10, 38, 39).

Together, molecular clock dysregulation and impaired ipRGC-mediated photic input may contribute to circadian disturbances affecting sleep, melatonin secretion, systemic blood-pressure rhythms, mitochondrial metabolism, and inflammatory activity. These changes could, in turn, exacerbate vascular and metabolic stress, creating a potentially bidirectional cycle that further increases optic nerve vulnerability. However, most supporting evidence derives from POAG, experimental glaucoma, or other retinal diseases, and direct evidence for a causal role of circadian dysregulation or ipRGC dysfunction in NTG remains limited. These mechanisms should therefore currently be considered biologically plausible contributors to NTG-related RGC vulnerability rather than established NTG-specific pathogenic pathways. Further longitudinal studies integrating circadian molecular profiling, ipRGC structural and functional assessment, and NTG progression are required to clarify their mechanistic relevance (10, 39–41).

## 3 Emerging gene therapies for NTG

### 3.1 AAV-based gene therapy for NTG

Adeno-associated virus (AAV) vectors are promising platforms for ocular gene delivery because of their relatively low immunogenicity, durable transgene expression, and capacity for retinal neuronal targeting (42). In NTG, AAV-based gene augmentation may provide IOP-independent neuroprotection by enhancing RGC resilience against neurotrophic deficiency, mitochondrial dysfunction, oxidative stress, neuroinflammation, and axonal degeneration. AAV2-mediated augmentation of the BDNF/TrkB pathway has shown sustained RGC and axonal protection in experimental glaucoma, whereas restoration of *OPTN*-associated mitochondrial transport through co-expression of *OPTN*, *TRAK1*, and *KIF5B* provides a more mechanism-directed approach linking NTG genetic susceptibility to mitochondrial and axonal dysfunction (43, 44). Other AAV-based strategies targeting apoptosis, oxidative stress, and axonal degeneration, including *EPO*, *NMNAT2*, and *SIRT6*, further support the potential of gene augmentation for neuroprotection. Nevertheless, current evidence remains predominantly preclinical, with limited NTG-specific validation, and challenges including RGC-selective transduction, vector capacity, immunogenicity, long-term transgene control, and molecular heterogeneity must be addressed before clinical translation (45–47).

### 3.2 RNA-based therapeutic strategies

#### 3.2.1 siRNA-mediated gene silencing

siRNA-mediated gene silencing provides a sequence-specific strategy for modulating pathogenic pathways in glaucoma. Experimental studies have shown that siRNAs targeting inhibitor of kappa B kinase (IKK) or the *β*2-adrenergic receptor can reduce IOP, whereas silencing Caspase-2, -3, -6, or -8 protects RGCs from apoptosis. In addition, Shp2-targeting siRNA delivered by AAV2 can reduce RGC apoptosis by modulating BDNF/TrkB-related signaling, while non-viral delivery of NogoR-1 siRNA promotes axonal growth, highlighting the potential of siRNA to target both neuronal survival and regenerative pathways. These findings are particularly relevant to NTG, in which apoptosis, impaired neurotrophic signaling, and axonal degeneration may contribute to progressive RGC loss independently of elevated IOP. Nevertheless, current evidence is predominantly preclinical, and the applicability of these targets to clinically defined NTG remains uncertain. Transient silencing, limited stability, and inefficient RGC-specific delivery remain major barriers, underscoring the need for optimized delivery systems and molecularly guided target selection (48–52).

#### 3.2.2 shRNA-mediated gene silencing

Short hairpin RNA (shRNA) is a vector-encoded RNA interference strategy that enables sustained, sequence-specific gene silencing through intracellular processing into siRNA-like molecules, offering more durable effects than synthetic siRNA (53). In experimental glaucoma and optic nerve injury models, shRNA-mediated silencing of intrinsic regenerative inhibitors, including components of the RhoA/ROCK, PTEN/mTOR, and JAK/STAT pathways, as well as transcriptional regulators such as KLF9 and Shp2, has been reported to enhance RGC survival and/or axonal regeneration, particularly when combined with neurotrophic or complementary pathway-modulating strategies (48).

#### 3.2.3 miRNA-based regulation

MicroRNAs (miRNAs) are endogenous non-coding RNAs that post-transcriptionally regulate multiple target genes and signaling pathways, enabling coordinated modulation of complex biological networks. This characteristic makes miRNA-based therapy particularly relevant to NTG. Several miRNAs have demonstrated neuroprotective effects in experimental glaucoma. For instance, miR-200a has been reported to attenuate RGC injury by modulating the FGF7/MAPK pathway and reducing glial activation and apoptosis (54). Other miRNAs, including miR-29b, miR-27a, miR-124, miR-141, miR-182, and miR-26a, have been implicated in the regulation of oxidative stress, extracellular matrix remodeling, inflammatory signaling, and neuronal survival, whereas miR-7, miR-93, and miR-210 may promote oxidative injury or apoptosis and thus represent potential inhibitory targets. Accordingly, therapeutic strategies may employ miRNA mimics to restore protective miRNAs or antagomiRs to suppress pathogenic miRNAs. By simultaneously modulating multiple pathways, miRNA-based interventions may enhance RGC resilience to IOP-independent stress and complement conventional IOP-lowering therapy (55–58). Nevertheless, most current evidence derives from POAG or experimental retinal injury models, and direct evidence in NTG remains limited. Further studies are therefore required to establish NTG-specific therapeutic efficacy, optimize ocular and RGC-selective delivery, and minimize off-target effects associated with the pleiotropic nature of miRNA regulation.

#### 3.2.4 Therapeutic mRNA delivery

Messenger RNA (mRNA)-based therapy enables transient, non-integrating expression of therapeutic proteins, providing greater temporal control than DNA- or AAV-based approaches. Unlike RNA interference, mRNA therapy restores or enhances protein expression and may therefore support neuroprotection, antioxidant defense, or mitochondrial homeostasis in genetically heterogeneous NTG. Preclinical studies have demonstrated lipid-mediated delivery of Cas9 mRNA and guide RNA to the trabecular meshwork, enabling transient *MYOC* editing and reversal of *MYOC*-associated ocular hypertension while limiting prolonged nuclease exposure (59, 60). However, these findings derive primarily from experimental glaucoma and *MYOC*-associated POAG models. Although mRNA could potentially be adapted for RGC-targeted neuroprotection or transient delivery of genome-editing components, its efficacy and safety for IOP-independent therapy in NTG remain to be established.

### 3.3 CRISPR/Cas-based genome editing

CRISPR/Cas-based genome editing enables targeted modification of disease-associated genes and may provide an IOP-independent strategy for protecting RGCs. In experimental glaucoma, genetic disruption of *SARM1*, a key mediator of programmed axonal degeneration, preserves RGC somata and axons and restores NAD⁺ homeostasis, while RGC-targeted deletion of the endoplasmic reticulum stress mediators *ATF4* or *DDIT3/CHOP* enhances RGC and axonal survival without directly affecting IOP (61). These findings highlight the potential of genome editing to target molecular drivers of neurodegeneration relevant to NTG. RGC-selective promoters such as mSncg may further improve cellular specificity and limit unintended editing in non-neuronal retinal cells.

However, direct evidence for CRISPR/Cas-based therapy in NTG remains limited, as most studies derive from experimental glaucoma, acute optic nerve injury, or genetically defined POAG models. Permanent genome modification also raises concerns regarding off-target effects, genomic instability, immunogenicity, and long-term safety. Future studies should focus on NTG-relevant pathways, including mitochondrial dysfunction, oxidative stress, neuroinflammation, axonal degeneration, and vascular dysregulation, while optimizing RGC-selective delivery and high-fidelity editing systems (Figure 1) (60–62).

**Figure 1:**
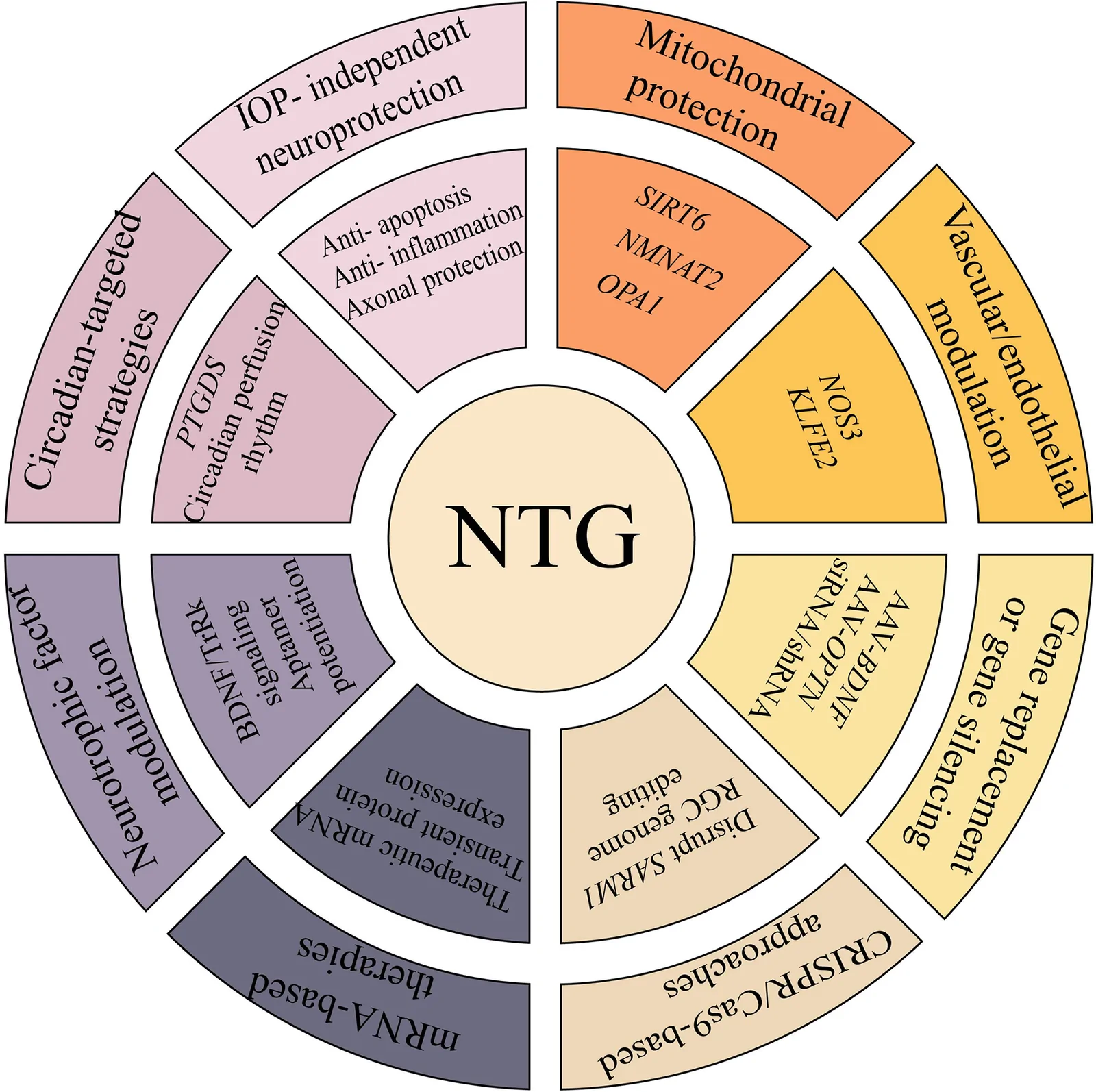
Therapeutic strategies targeting interconnected pathogenic pathways in normal-tension glaucoma.

## 4 Aptamer-based targeted therapy and delivery

Aptamers are short single-stranded DNA or RNA oligonucleotides that adopt specific three-dimensional structures to selectively recognize proteins, receptors, nucleic acids, and cell populations. Their small size, chemical accessibility, facile modification, and potentially low immunogenicity make them attractive molecular targeting ligands (57–60). Generated primarily through SELEX-based approaches, including cell-, tissue-, and *in vivo*-SELEX, aptamers can be optimized for selective cellular or tissue targeting and chemically modified to improve stability and pharmacokinetic properties. In NTG, aptamers may facilitate receptor-mediated delivery of therapeutic cargos through direct conjugation or incorporation into nanocarriers and are particularly compatible with siRNA, miRNA, and mRNA platforms, enabling cell-selective gene modulation or transient therapeutic protein expression (63–65).

Given the central role of RGC degeneration in NTG, RGC-selective aptamers could potentially enhance the delivery of neuroprotective cargos, such as BDNF mRNA or regulatory siRNAs and miRNAs targeting pathways involved in oxidative stress, neuroinflammation, apoptosis, and axonal degeneration. Aptamers targeting vascular or inflammatory components may also enable modulation of non-neuronal mechanisms contributing to disease progression. However, RGC-targeted aptamer therapy remains largely conceptual, and its clinical translation requires the identification of specific RGC surface markers and validation of targeting efficiency, cellular internalization, stability, safety, and therapeutic efficacy in chronic NTG-relevant models. Thus, aptamers may serve primarily as a precision-targeting platform to enhance cell-specific, IOP-independent therapeutic delivery in NTG rather than as a standalone treatment (63, 66).

## 5 Discussion

NTG represents a distinct clinical phenotype of glaucomatous optic neuropathy characterized by progressive RGC and axonal degeneration despite statistically normal IOP. Although IOP reduction remains the only established disease-modifying treatment, continued progression in some patients despite adequate IOP control indicates that intrinsic optic nerve susceptibility and IOP-independent mechanisms contribute substantially to disease progression. In this review, we integrate current evidence supporting a multidimensional model of NTG pathogenesis encompassing vascular dysregulation, mitochondrial dysfunction and oxidative stress, autophagy–neuroinflammation, and axonal transport abnormalities, with circadian regulation emerging as a potential integrative mechanism. Importantly, these pathways are not necessarily unique to NTG; rather, their interaction may increase RGC vulnerability under normal-range IOP and thereby contribute to the characteristic phenotype of NTG.

A major objective of this review was to distinguish NTG-specific genetic evidence from mechanisms shared with other glaucoma subtypes or inferred from experimental models, addressing an important limitation of previous reviews. Among the genes discussed, *OPTN* and *TBK1* currently have the strongest genetic evidence for NTG susceptibility, whereas *NOS3* provides a potential genetic link between endothelial dysfunction and impaired optic nerve perfusion. In contrast, *MYOC*, *LTBP2*, and *LOXL1* are primarily established in POAG, developmental glaucoma, or pseudoexfoliation glaucoma and are largely associated with IOP regulation and extracellular matrix remodeling; their contribution to NTG remains limited or indirect. Similarly, targets such as *BDNF*, *SARM1*, *DLK/MAP3K12*, *OPA1*, *SIRT1*, *SIRT6*, and *NMNAT2* are supported mainly by experimental glaucoma, optic nerve injury, or broader neurodegenerative studies rather than NTG-specific human genetic evidence. We therefore classified candidate genes according to the source and strength of evidence, their relationship to IOP, and their potential relevance to RGC vulnerability in NTG (Table 1), thereby avoiding the assumption that all glaucoma-associated genes are NTG-specific.

**Table 1:** Evidence-stratified classification of candidate genes and molecular targets in NTG.

Gene/ Target	Biological pathway	Evidence specifically relevant to NTG	Evidence in other glaucoma subtypes	Evidence level^a^	IOP dependence	Proposed relevance to NTG	Therapeutic potential	References
*OPTN*	Autophagy/mitophagy	NTG-associated variants	POAG/glaucoma	I + III	Independent	RGC degeneration	Autophagy/neuroprotection	(28)
*TBK1*	Autophagy/mitophagy	NTG-associated CNVs/variants	Glaucoma	I + III	Independent	Mitochondrial/RGC vulnerability	*TBK1*/autophagy modulation	(29)
*NOS3*	NO/vascular regulation	NTG-associated polymorphisms	Vascular/glaucoma phenotypes	I	Independent	ONH hypoperfusion	NO/endothelial modulation	(14)
*KLF2*	Shear stress/endothelial homeostasis	Limited direct evidence	Vascular biology	V	Independent	Vascular autoregulation	Vascular modulation	(15)
*LTBP2*	ECM/microfibrils	Limited evidence	PCG/ECM-related glaucoma	IV	Mainly dependent	ECM remodeling; possible vascular effects	ECM/TGF-*β* modulation	(15, 16)
*LOXL1*	Elastic fiber/ECM	Limited evidence	PEX/PEXG	IV	Mainly dependent	Ocular biomechanics; possible vascular effects	ECM modulation	(15, 16)
*BDNF*	TrkB/neurotrophic signaling	No direct NTG evidence	Experimental glaucoma	III/IV	Independent	RGC survival	BDNF/mRNA/aptamer therapy	(43)
*SARM1*	Axonal degeneration	No direct NTG evidence	Experimental glaucoma/injury	III	Independent	Axonal degeneration	*SARM1* inhibition	(61)
*DLK/MAP3K12*	Axonal stress/JNK	No direct NTG evidence	Experimental glaucoma/injury	III	Independent	RGC degeneration	*DLK* inhibition	(61)
*OPA1*	Mitochondrial dynamics	Limited evidence	Experimental glaucoma	III	Independent	Mitochondrial dysfunction	Mitochondrial protection	(25)
*SIRT1*	Mitochondrial/oxidative stress	Limited evidence	Experimental models	III	Independent	Oxidative stress resistance	Sirtuin modulation	(46)
*SIRT6*	Stress response/mitochondria	Limited evidence	Experimental models	III	Independent	Metabolic/oxidative protection	*SIRT6* modulation	(47)
*NMNAT2*	NAD⁺/axonal homeostasis	No direct NTG evidence	Axonal injury/glaucoma models	III	Independent	Axonal metabolic resilience	NAD⁺/*NMNAT2* modulation	(67)
mtDNA variants	Oxidative phosphorylation	NTG molecular alterations	POAG/neurodegeneration	II	Independent	Mitochondrial dysfunction	Mitochondrial protection	(18, 23)
*PTGDS*	Prostaglandin/vascular/inflammatory signaling	Limited evidence	Glaucoma and neurovascular studies	III/IV	Potentially independent	Vascular regulation, neuroinflammation and RGC vulnerability	*PTGDS*/prostaglandin pathway modulation	(35, 36)

a Evidence levels: I, genetic association studies in NTG patients; II, differential expression or molecular studies using NTG-derived tissues/biospecimens; III, experimental glaucoma, optic nerve injury, cellular or animal models; IV, evidence primarily extrapolated from POAG or other glaucoma subtypes; V, mechanistically plausible but currently lacking direct NTG-specific human or experimental evidence.

A further strength and novelty of this review is the integration of vascular, mitochondrial, and neuronal mechanisms into a unified framework, rather than considering these processes as independent pathogenic pathways. Vascular dysregulation and impaired ONH perfusion may reduce RGC metabolic reserve and increase susceptibility to mitochondrial and oxidative stress, while defective mitochondrial quality control, axonal transport, and chronic inflammation may further reinforce neuronal injury. Circadian dysregulation may provide an additional regulatory layer linking vascular, metabolic, and immune processes. This interconnected framework may help explain the progression of RGC degeneration despite normal-range IOP and the biological heterogeneity of NTG.

A second contribution is the systematic evaluation of emerging gene- and nucleic-acid-based strategies for IOP-independent neuroprotection, including AAV-mediated gene delivery, siRNA, shRNA, miRNA, mRNA, CRISPR/Cas-based genome editing, and aptamer-mediated targeting. These approaches offer opportunities to modulate pathogenic pathways, enhance neuroprotective signaling, and improve cellular specificity beyond conventional IOP lowering. However, most evidence remains preclinical and derives from experimental glaucoma or optic nerve injury models rather than well-characterized NTG populations. RGC-selective delivery, therapeutic duration, off-target effects, and long-term safety remain major challenges for clinical translation.

Given the multifactorial nature of NTG, future therapy will likely require mechanism- and phenotype-guided strategies rather than a single universal target. Integrating IOP reduction with modulation of vascular, mitochondrial, inflammatory, and neuronal pathways, together with human genetics, longitudinal phenotyping, ocular perfusion assessment, multimodal imaging, and multi-omics profiling, may facilitate identification of biologically distinct NTG subgroups and actionable vulnerabilities. Importantly, distinguishing NTG-specific determinants from mechanisms shared with other glaucoma subtypes will be critical for translating emerging gene and RNA technologies into effective precision therapies for patients with progressive disease despite apparently controlled IOP.
